# Uncommon Presentation of a Pulmonary Hydatid Cyst: A Case Report

**DOI:** 10.7759/cureus.79830

**Published:** 2025-02-28

**Authors:** Ahmed Maalej, Ali Al Janaahi, Khalid E Attia

**Affiliations:** 1 Clinical Sciences Department, College of Medicine, University of Sharjah, Sharjah, ARE; 2 Department of Radiology, Emirates Health Services, Sharjah, ARE

**Keywords:** echinococcus antibody, echinococcus granulosus, hydropneumothorax, pulmonary hydatid cyst, pyopneumothorax, ruptured hydatid cyst, tree-in-bud pattern

## Abstract

Pulmonary hydatid cysts are rare entities, accounting for a small fraction of radiological findings. This case report presents a 43-year-old male with an atypical presentation initially suggestive of pneumonia. During a 13-day hospital admission, the patient’s condition deteriorated, and further investigations revealed a complicated ruptured hydatid cyst. Definitive management included thoracotomy, cyst removal, and lung decortication. Patient's postoperative course was uneventful. Laboratory tests confirmed the diagnosis with positive *Echinococcus* antibody serology. The patient was discharged in stable condition with four and a half months of albendazole therapy and a pulmonologist follow-up plan. This report highlights the diagnostic challenges, clinical course, and surgical management of a ruptured pulmonary hydatid cyst.

## Introduction

This case report outlines the clinical presentation, diagnostic journey, treatment, and follow-up of a 43-year-old male presenting with pneumonia-like symptoms, ultimately diagnosed with a pulmonary hydatid cyst. Hydatid cysts are caused by the parasitic tapeworm Cystic echinococcosis, which can be found in the liver and lungs containing severely allergenic fluid. Hydatid cysts in the lungs are less common than hepatic cysts but are still significant, particularly in endemic regions. According to the annual epidemiological report for 2022 by the European Center for Disease Prevention and Control, there have been 731 confirmed echinococcosis cases in the European Union in 2022 [1]. The World Organisation for Animal Health has calculated the infection rate to be 200 per 100,000 people who are in close contact with their domestic dog in a rural population [2]. This is in comparison to one per 100,000 in a normal population [2]. This condition starts through the ingestion of parasite eggs from infected animals and is more prevalent in sheep-rearing regions [3-5]. It is important to note that the presenting complaint in the Echinococcus hydatid cyst depends on the cyst location. The disability-adjusted life year for this condition has been estimated at around 285,500 years [6]. The majority of infections occur in the liver, followed by the lung [3-5]. It has been observed in patients with acquired immunodeficiency syndrome (AIDS) an accelerated growth in cyst size, which suggests that immune suppression may play a role in prognosis [6]. Within the last couple of years, there has been a rise in cases in Europe and North America, which has been linked to immigration [7]. Researchers are urgently requesting the development of a new medication and vaccine [6]. 

In Brunetti et al., the majority (80%) of cases form a solitary cyst in one organ [8]. The fluid released from a hydatid cyst is antigenic, which explains the occurrence of anaphylaxis, asthma, membranous nephropathy, and urticaria in a patient with no family history of such conditions [9]. In respiratory-specific cases, such as in our case, we require additional research into non-invasive procedures; however, according to current research, the average hepatic hydatid cyst patient admission at a healthcare facility drops to 4.2 days from the average of 12.7 in open surgery due to the rise of ultrasound-guided percutaneous drainage which reduces side effects, the incidence of hospital-acquired pneumonia and healthcare burden, and opens more beds for those in need [4]. The high false negative results further complicate the early establishment of a diagnosis, which has been estimated to be up to 20% of hepatic cyst patients and 40% of respiratory cyst patients that present to the hospital with negative specific serum antibodies (IgG) [9]. Our case report will help raise awareness of this infection, echinococcosis, as one of the 17 most neglected infections in tropical areas, according to the WHO [3,6].

## Case presentation

Clinical history

A 43-year-old male, a Syrian national and a carpenter by trade, presented to the emergency department with a four-day history of fever, productive sputum, dyspnea, and right-sided chest pain. The patient reported two episodes of vomiting and four episodes of diarrhea in the 24 hours preceding his admission. His medical history included an on-and-off mild erythematous itchy rash that started a month ago, present over the abdomen, thighs, groin, and armpits, diagnosed as tinea corporis by a dermatologist and initially treated using a 200 mg daily oral Itraconazole, 2% ketoconazole and 2% fusidic acid cream. He denied any prior history of tuberculosis, smoking, or alcohol consumption. The patient's medical history was insignificant.

Examination and laboratory findings

A physical examination of the patient revealed a conscious patient, oriented to time, place, and person, with stable vitals except for a fever of 39.5°C. The cardiovascular, abdominal, and CNS examinations were unremarkable. The respiratory examination revealed decreased air entry, occasional crackles, and dullness on percussion at the base of the right lung. A mild rash was noted on the abdomen, thighs, groin, and armpits. During the patient's stay in the emergency room and the inpatient ward, standard and specific blood investigations were conducted. These revealed elevated levels of C-reactive protein (CRP), erythrocyte sedimentation rate (ESR), white blood count (WBC), neutrophil percentage, Echinococcus antibody, and pleural fluid lactate dehydrogenase (LDH), as shown in Table 1 below.

**Table 1:** Laboratory Findings Bold text signifies values outside the reference range. Lab investigation revealed an elevated WBC, neutrophil percentage, Echinococcus antibody and pleural fluid lactate dehydrogenase (LDH).

Investigation:	Recorded Value	Unit	Reference Range
Red Blood Cell Count (RBC)	4.29	x10^12/L	4.2 - 5.9
Hemoglobin (Hb)	13	g/dL	12.0 - 16.0
Hematocrit (Hct)	37.7	%	37.0 - 47.0
Mean corpuscular volume (MCV)	87.9	fL	80.0 - 100.0
Mean corpuscular hemoglobin (MCH)	30.3	pg	27.0 - 33.0
Mean corpuscular hemoglobin concentration (MCHC)	34.5	g/dL	32.0 - 36.0
Red cell distribution width (RDW)	13.4	%	11.5 - 14.5
Platelet Count	239	x10^9/L	150 - 450
Mean platelet volume (MPV)	9.9	fL	7.5 - 11.5
White Blood Cell Count (WBC)	22.52	x10^9/L	4.0 - 11.0
Neutrophil %	87	%	40 - 70
Lymphocyte %	7.8	%	20 - 40
Monocyte %	4.2	%	2 - 8
Eosinophil %	0.8	%	1 - 4
Basophil %	0.1	%	0 - 1
C-reactive protein (CRP)	250	mg/L	0 - 10
Erythrocyte sedimentation rate (ESR)	59	mm/Hr	≤15
D-dimer	1.51	mg/L	0 - 0.5
Total Protein	62	g/L	60 - 80
Albumin	24	g/L	35 - 50
Echinococcus antibody	>1:2560		<1:160
Pleural fluid LDH	2119	u/L	<200

Imaging studies

**Figure 1:**
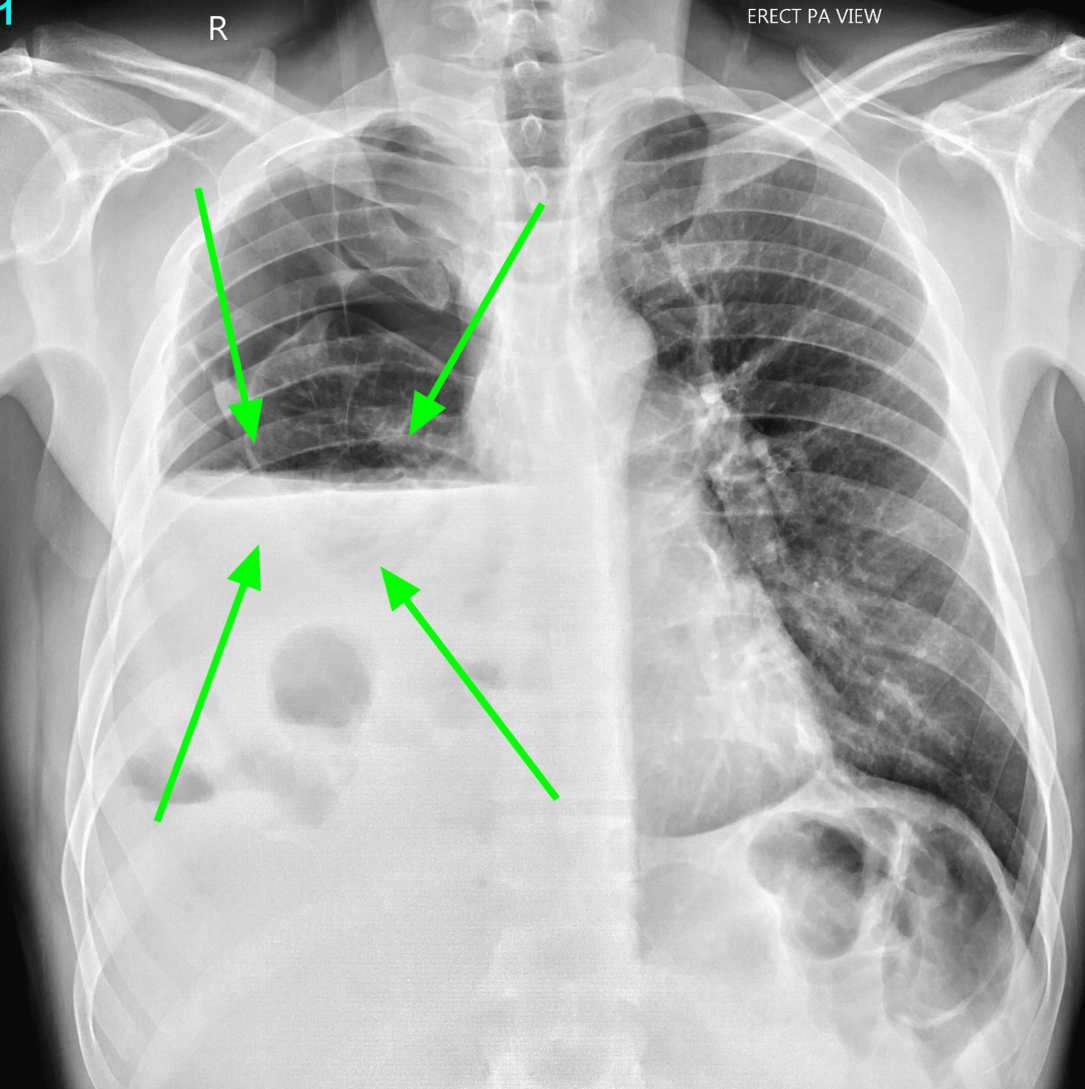
Chest X-ray taken on day one revealed right hydropneumothorax with subsequent partial right lung collapse. The green arrow points to the right-sided hydropneumothorax.

**Figure 2:**
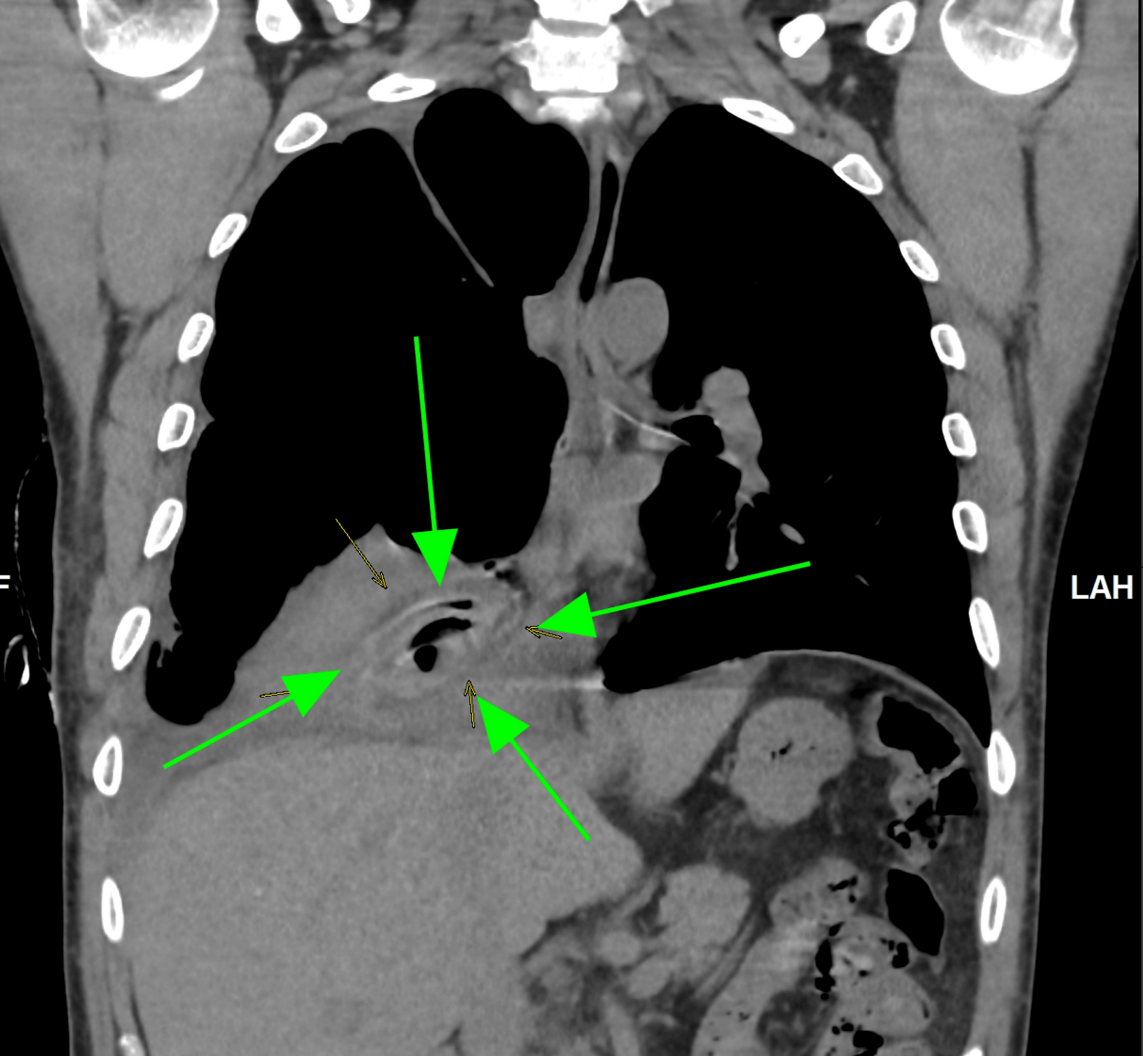
A CT scan (coronal) taken on day four revealed an ovoid lesion (green arrow) right lung lower lobe anterior basal segment using the soft-tissue window. The green arrow points to the large ovoid lesions in the right lower lobe, which happen to be the right lung hydatid cyst in question.

**Figure 3:**
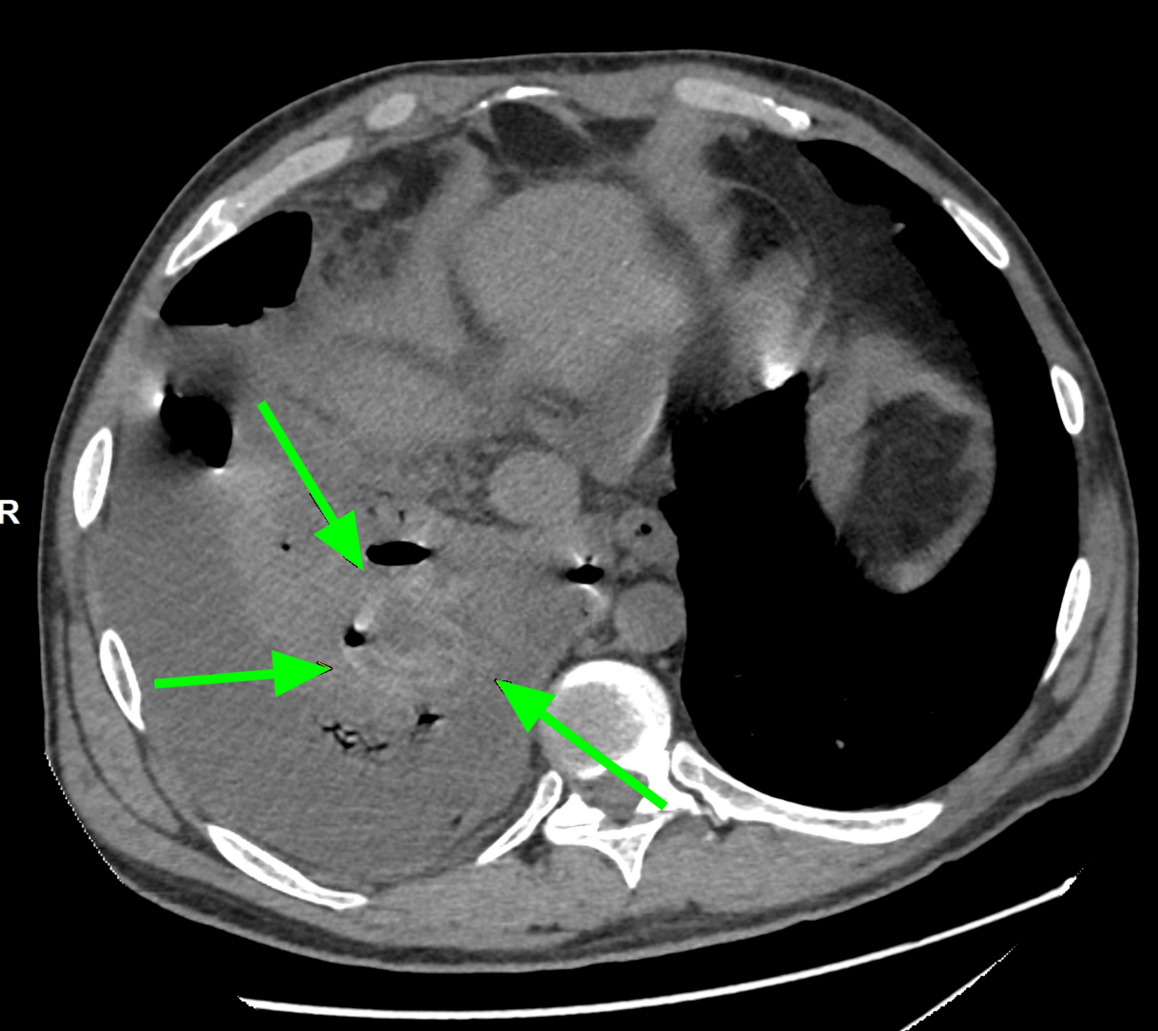
A CT scan (axial) taken on day four revealed an ovoid lesion (green arrow) right lung lower lobe anterior basal segment using the soft-tissue window. The green arrow points to the large ovoid lesions in the right lower lobe, which happen to be the right lung hydatid cyst in question.

**Figure 4:**
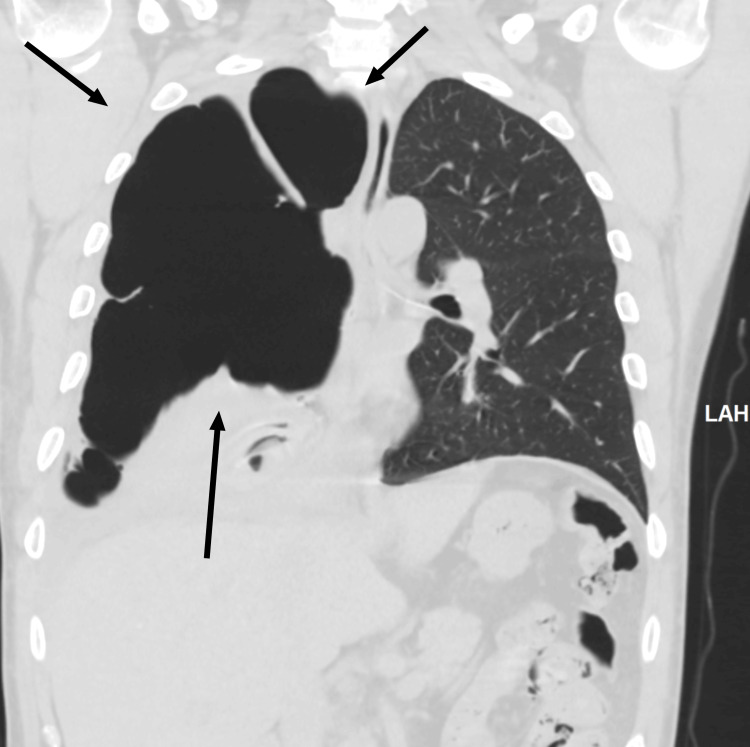
Day four CT scan (coronal) using the lung window to visualize the right lung. The black arrow points to the right lung. The dark black color occupying the right lung describes the loss of the lung parenchyma and the presence of air.

**Figure 5:**
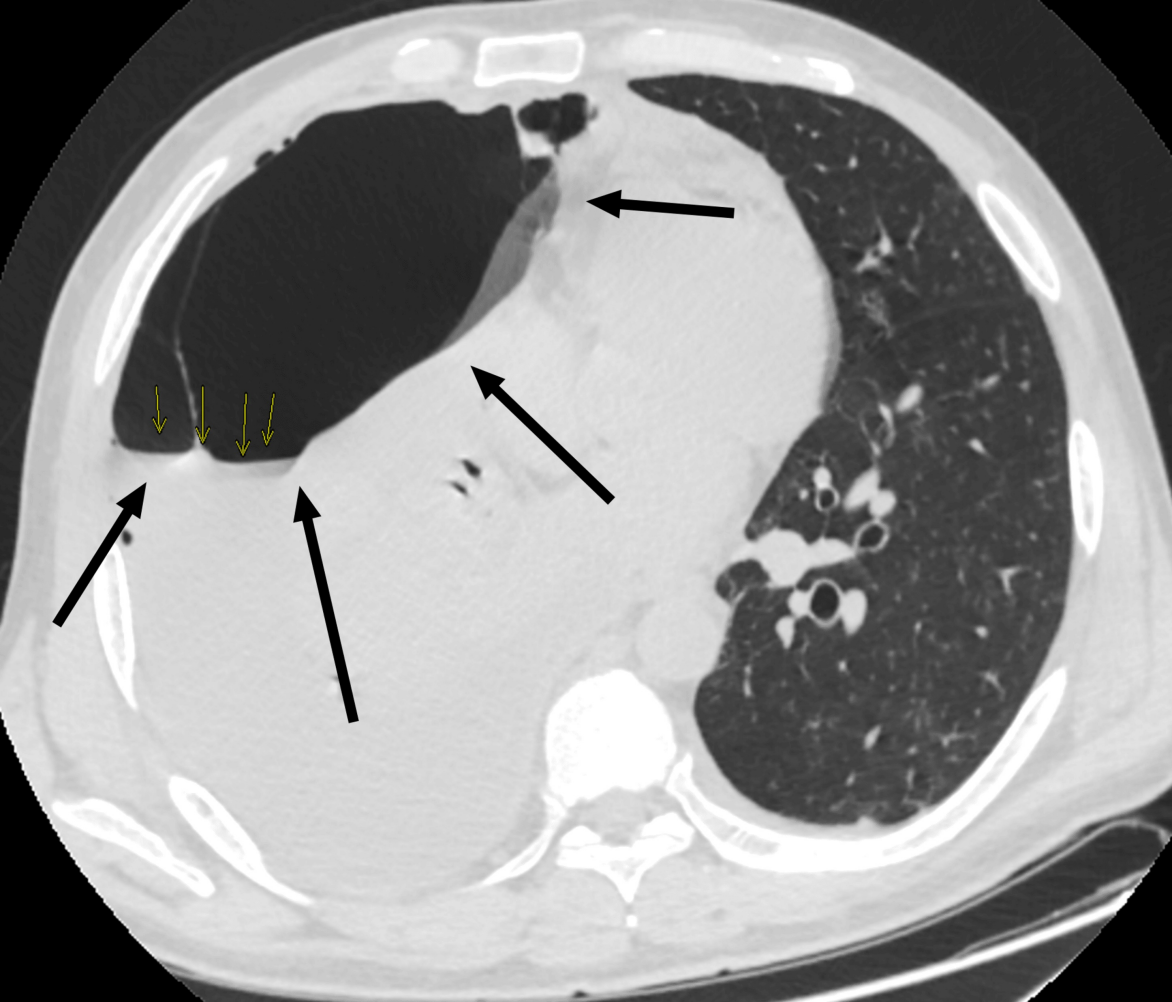
Day four CT scan (axial) using the lung window to visualize the right lung. The black arrow points to the right lung. The dark black color occupying the right lung describes the loss of the lung parenchyma and the presence of air.

The emergency doctor initially requested a portable chest X-ray. The chest X-ray, as seen below in Figure 1, showed a massive right-sided hydropneumothorax with air-fluid levels and partial lung collapse. During the patient's admission to the hospital, a computerized tomography (CT) scan of the chest was done, as seen below in Figure 2 and Figure 3, which showed moderate hydropneumothorax with loculations of a large ovoid lesion in the right lower lobe on a soft tissue window. Loss of the lung parenchyma, the presence of air, and the tree-in-bud pattern indicating endobronchial spread of infection in the left lung were also viewed on a computerized tomography (CT) scan, as seen below, using the lung window in Figure 4 and Figure 5.

Management

**Figure 6:**
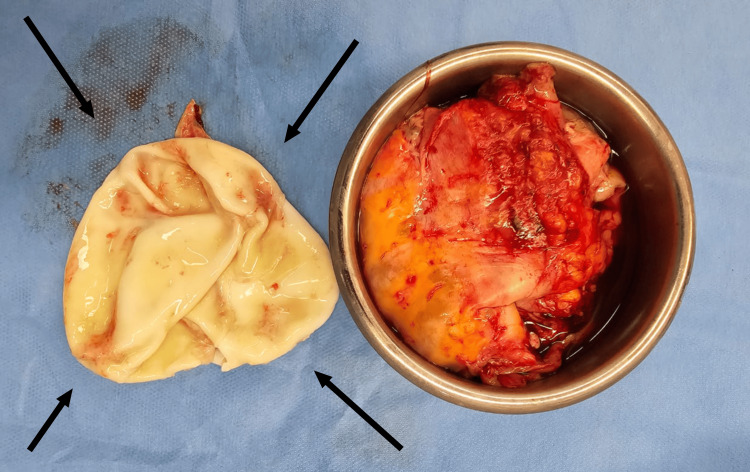
Post-operation, the hydatid cyst (left) was removed by thoracic surgery.

A right intercostal 28f drain was inserted, evacuating 1500 ml of dark fluid with air. The patient's condition worsened, necessitating a thoracotomy, cyst excision, and lung decortication. Postoperative management included pain control (initially with epidural analgesia via an epidural catheter and controlled by the patient pump inserted at the level between T8 and T9, with 0.125% bupivacaine 4 ml/hour for the first three days, later replaced by oral tramadol) and 400 mg oral twice daily albendazole therapy. Secondary infections were managed with 4.5 gram IV every eight hours of piperacillin/tazobactam as per culture sensitivity report, and pleuritic pain was controlled with 600 mg oral Ibuprofen four times daily. Figure 6 below depicts the removed cyst. During the patient's stay at the hospital, he did not suffer from any secondary infections. 

Pathology and outcome

**Figure 7:**
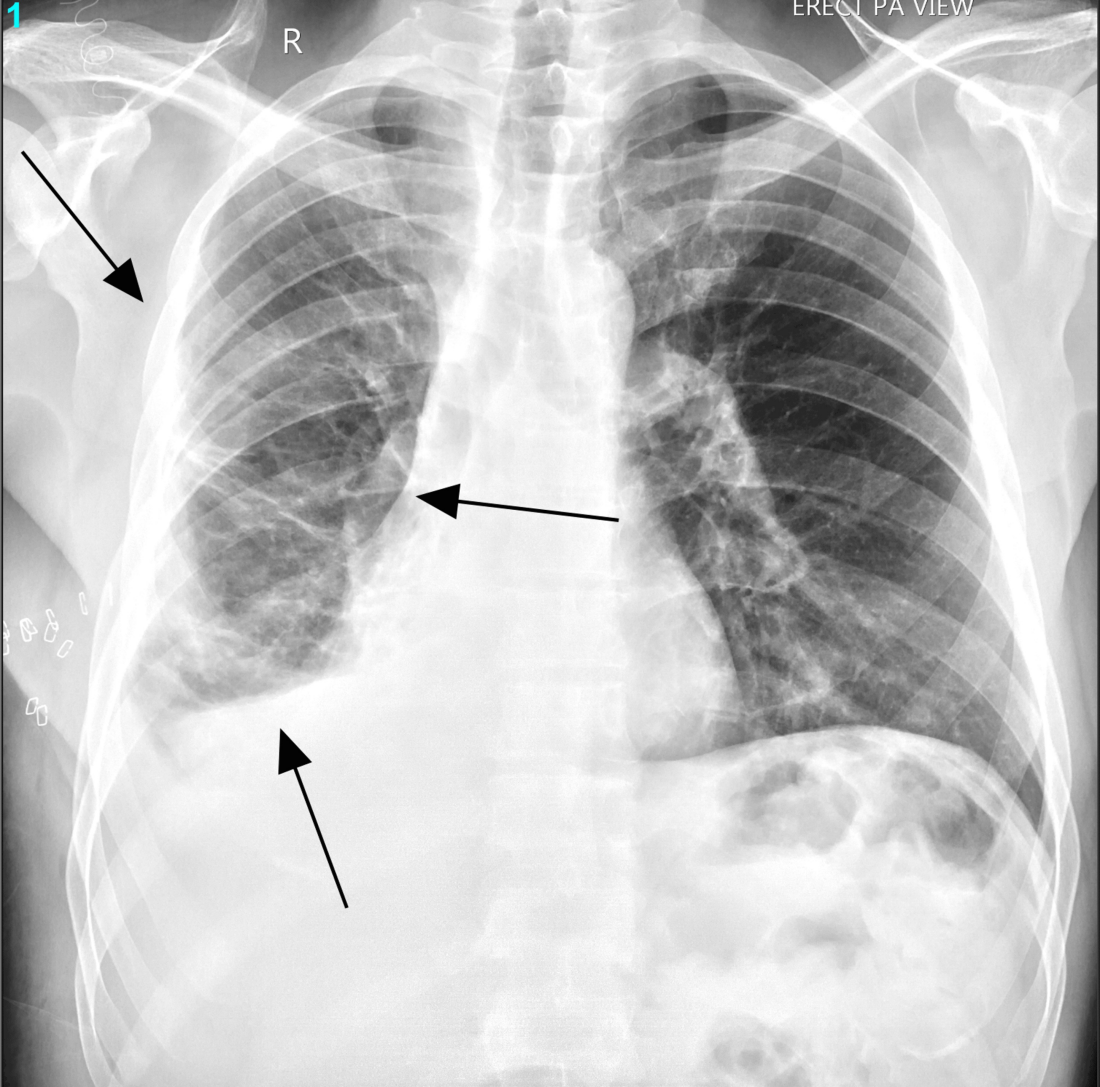
X-ray taken prior to patient discharge. Compare the right lung (arrows) to Figure 1, which was taken in the emergency room.

Gross pathology revealed a 25 cm ruptured hydatid cystic with calcified walls and daughter cysts. Histological examination confirmed the diagnosis, showing laminated acellular cyst walls and chronic inflammatory infiltrates. Figure 7 below is an X-ray taken prior to the patient's discharge, depicting a radiological improvement. When the patient's clinical progress improved, with no pain, no fever, and no shortness of breath and he was alert, conscious, and comfortable, he was discharged after a 13-day stay in the hospital. He was placed on a four-and-a-half month post-discharge 400 mg oral twice daily albendazole therapy with pulmonary clinic follow-ups. Shortly after discharge, the patient presented to the pulmonary clinic with some complaints of fatigue but otherwise felt better compared to his discharge state. The patient was sadly non-compliant with his remaining monthly clinic follow-ups; therefore, it is impossible to assess his current state.

## Discussion

Epidemiology

Classically, infected protozoa echinococcus granulosus are seen in humans who are known to reside in sheep-raising regions, especially in close contact with infected dogs, infected livestock, contaminated water, and contaminated food [3-6]. In up to 70% of cases, the liver is the primary site of infection, whereas the lungs are up to 30% involved [4,6]. From the gastrointestinal tract, the echinococcus eggs hatch and release oncospheres, which can migrate to the liver through the portal system [6]. Echinococcus can also migrate directly to the lungs through the lymphatic ducts [3,5]. In the lung, these embryos are known to form cysts called hydatid cysts and, in most circumstances, are known to progress slowly; however, when it comes to the pediatric population, the cyst formation is accelerated [3]. The infection rate in children is greater than that of adults, possibly associated with the increased amount of time spent between children and the family pet [3,5]. Adults and other organs are still susceptible to infection. In children, the disease is often associated with delayed or stunted growth [3]. The diagnosis is more common in men due to the difference in the workplace compared to women [3]. In endemic areas, a simple cyst with a thick wall may warrant further investigation [3]. The average patient remains asymptomatic anywhere between five to 20 years until they become symptomatic [3,6].

Clinical features

Patients are asymptomatic when the cyst is small and intact [3]. As time passes, the development and disintegration of daughter cysts and blood capsules release free hooklets into circulation [3]. The average cyst enlarges at a rate of 1 to 50 mm per year [8]. Patients become symptomatic when cysts enlarge and occupy >70% of the target organ or, in the case of the liver, enlarge to greater than 10 cm, compress surrounding structures, and rupture, with the development of secondary infections [3,5,6]. Patients can present with complaints that may vary based on the size and site of infection [3,5]. For example, liver involvement may lead to presentations of abdominal discomfort and poor appetite, while pulmonary involvement would lead to symptoms such as cough and hemoptysis [3,5,6]. When a lung cyst ruptures, such as in our case, the patient may present with generalized symptoms such as fever, malaise, urticaria, eosinophilia, anaphylactic shock, and respiratory-specific complaints such as cough, hemoptysis, and chest pain mimicking pneumonia [3,5,6]. When an infection spreads to the lung, the lower lobes are the most commonly infected, such as in our case [3]. Almost half of the pulmonary hydatid cysts result in rupture, and an average of 4% of cases result in a pneumothorax, such as in our case [7]. This is commonly triggered when the patient is exposed to physical trauma, severe cough, or repeated sneezing [3]. As mentioned previously in the introduction, the majority of infections and cysts involve a solitary organ [8]. The involvement of a distant secondary cyst typically occurs when cysts independently rupture subclinically or after exposure to physical trauma [9]. 

Diagnosis

Diagnosis by physical examination of the chest alone seldom occurs. However, it is important to remember that, in general, cysts present with dullness to percussion and fluid thrill [3]. It is also important to remember that chronic fever and weight loss, which are common with this disease, may mimic malignancy [3]. Diagnoses can occur when vomit, urine, and stool demonstrate echinococcus scolices and hooklets when examined histologically [3]. Rarely, a patient may cough the cyst's contents, commonly referred to as coughing grape skins, which is also considered diagnostic, however this is a rare finding [3]. Deranged liver function tests, IgE, and eosinophilia are seen in half of the patients diagnosed with hydatid cysts; however, elevated IgE alone in endemic areas is not specific for echinococcus and is, therefore, an unreliable method to investigate for echinococcus [3,6]. While still in the research phase, echinococcus antigen detection seems to be promising compared to the antibody blood test [6]. As in our case, diagnoses are typically established through proper history, physical examination, laboratory investigations, and radiology. 

Hydatid cysts have often been discovered and diagnosed through incidental chest radiological investigations. As in a third of these cases, the patients are asymptomatic with an intact cyst [3]. On chest X-ray, cysts could be unilateral or bilateral, located in the hilum, central, or periphery, or a combination of different locations. These cysts are homogeneous oval to round smooth surface structures with no calcifications [3,7]. Assessment of the cyst is mainly done through ultrasound, and the classification is used for staging of the disease as well as treatment decisions. The disease can be classified into three categories, which are active, transitional, and inactive. When ultrasound assessment cannot be done, computed tomography (CT) scan and magnetic resonance imaging are done to stage the disease [3]. On CT, the cyst would present as round opaque lesions, and in some cases where the cyst communicates, wall defect and passage of the cyst contents through the defect may be observed [7]. It could also be discovered incidentally during bronchoscopy when a large asymptomatic cyst causes a bronchial displacement [3]. The highest rate of re-infection is when a cyst ruptures into the bronchus [7]. Early conduction of imaging modalities, such as ultrasound, would aid in narrowing differentials with its superior ability to differentiate fluid cysts from solid tumors [3], as it is highly specific and sensitive [3]. It is also considered pathognomonic when an ultrasound reveals multiple daughter cells with a honeycomb appearance [3]. Differential diagnoses of pulmonary hydatid cysts include lung abscess, hamartoma, pulmonary arteriovenous fistula, granuloma, malignant tumor, and metastasis [3]. 

Management

While low, there exists a chance of patients spontaneously recovering from pulmonary hydatid cysts by coughing out the cyst with its contents during its early stages of formation [7]. If left without healthcare, two-thirds of patients will expire from this condition [3]. With modern medical treatment, the fatality rate is approximately to be around 3% [6]. Lung tissue is known to heal rapidly and with regulated recovery; therefore, early treatment is recommended [3]. Previously, antihelminth medications along with surgery were the only means of treatment; however, with ultrasound-guided percutaneous drainage, a procedure abbreviated as PAIR could be performed, which stands for puncture, aspiration, injection, and then reaspiration of the cyst with saline [3,4,6]. Research has shown that within two years, percutaneous procedures are as similar in treatment as surgical cystectomy [4]. The surgical approach is associated with an increased incidence of fever and a sharp rise in antibodies revealed after surgery [4]. To prevent intrapulmonary communication, surgical intervention is preferred over medical due to the superior ability to remove endocyst, preventing rupture and expiration of cyst content [3]. 

Surgical removal is the gold standard, especially for complicated or ruptured cysts, with careful care not to puncture the cyst [4,6]. If the echinococcus antibody fails to drop after surgical removal, another cyst could present [3]. Preoperative albendazole simplifies removal and reduces intra-cystic pressure [3]. Adjunct albendazole therapy prevents recurrence and manages residual infection [3]. Percutaneous aspiration with albendazole is an alternative for inoperable cases [4]. Yearly ultrasound follow-up is required for the first five years due to high rates of relapse associated with surgical intervention and incomplete cure with percutaneous procedure [3,6]. The minimally invasive uniportal video-assisted thoracoscopic surgery (u-VATS) is the future approach to surgically treating pulmonary hydatid cysts that are less than 15 cm in size. It has shown that this surgical intervention has shorter surgery time, less intraoperative blood loss, shorter chest tube duration, lower chest tube drainage, reduced postoperative pain, fewer required painkillers postoperatively, better cosmetic outcomes, and reduced overall complications compared to a thoracotomy [10]. The future of this new surgical approach is uncertain as there are not enough patients that stimulate the increased demand required to increase physician training and the use of this procedure. 

## Conclusions

This case underscores the importance of considering hydatid cysts in patients from endemic regions presenting with atypical respiratory symptoms. Hydatid cysts are considered a significant health problem in India, Iran, China, and Mediterranean countries, which lack satisfactory environmental health, preventive medicine, and veterinarian services. The patient being from a Mediterranean country such as Syria and working in a blue-collar job is a typical match of the groups of people that are commonly infected with this organism. Echinococcosis continues to be a major community health burden in several countries, and in some terrains, it constitutes an emerging and re-emerging disease. Early recognition and timely surgical intervention are critical for preventing complications and ensuring recovery. Our patient was discharged healthy with regular follow-ups with the pulmonologist. However, it is impossible to determine his current medical state as he has not presented to his follow-up consultations other than the initial follow-up consultation. 
